# Transcriptomic and Metabolomic Analyses Reveal the Roles of Flavonoids and Auxin on Peanut Nodulation

**DOI:** 10.3390/ijms241210152

**Published:** 2023-06-15

**Authors:** Jianguo Wang, Ruining Diao, Zhengfeng Wu, Shubo Wan, Sha Yang, Xinguo Li

**Affiliations:** 1 Institute of Crop Germplasm Resources, Shandong Academy of Agricultural Sciences, Jinan 250100, China; wang_jianguo2020@163.com (J.W.); drn2485308419@163.com (R.D.); 2 Shandong Peanut Research Institute, Qingdao 266100, China; wzf326@126.com; 3 Shandong Academy of Agricultural Sciences, Jinan 250100, China; wansb@saas.ac.cn

**Keywords:** peanut, nodulation, flavonoid, auxin-response factor, ABC transporter

## Abstract

Rhizobia form symbiotic relationships with legumes, fixing atmospheric nitrogen into a plant-accessible form within their root nodules. Nitrogen fixation is vital for sustainable soil improvements in agriculture. Peanut (*Arachis hypogaea*) is a leguminous crop whose nodulation mechanism requires further elucidation. In this study, comprehensive transcriptomic and metabolomic analyses were conducted to assess the differences between a non-nodulating peanut variety and a nodulating peanut variety. Total RNA was extracted from peanut roots, then first-strand and second-strand cDNA were synthesized and purified. After sequencing adaptors were added to the fragments, the cDNA libraries were sequenced. Our transcriptomic analysis identified 3362 differentially expressed genes (DEGs) between the two varieties. Gene ontology and Kyoto Encyclopedia of Genes and Genomes analyses revealed that the DEGs were mainly involved in metabolic pathways, hormone signal transduction, secondary metabolic biosynthesis, phenylpropanoid biosynthesis, or ABC transport. Further analyses indicated that the biosynthesis of flavonoids, such as isoflavones, flavonols, and flavonoids, was important for peanut nodulation. A lack of flavonoid transport into the rhizosphere (soil) could prevent rhizobial chemotaxis and the activation of their nodulation genes. The downregulation of *AUXIN-RESPONSE FACTOR* (*ARF*) genes and lower auxin content could reduce rhizobia’s invasion of peanut roots, ultimately reducing nodule formation. Auxin is the major hormone that influences the cell-cycle initiation and progression required for nodule initiation and accumulates during different stages of nodule development. These findings lay the foundation for subsequent research into the nitrogen-fixation efficiency of peanut nodules.

## 1. Introduction

Nitrogen is an essential nutrient whose largest reservoir is the atmosphere, but atmospheric nitrogen (N_2_) is not available to most organisms. Leguminous plants form symbiotic relationships with rhizobia, bacteria that transform N_2_ into plant-available ammonia (NH_3_) within nodules formed on the roots of the legumes [1,2]. These symbioses contribute about 2145 tons of nitrogen to legume crops every year, accounting for 30–60% of the nitrogen accumulated by plants [3,4]. Compared with artificial nitrogen fertilizers, biologically fixed nitrogen is not readily leached or volatized into the environment, limiting the ecological problems arising from nitrogen pollution. Legumes are therefore an important factor in sustainable agricultural development and the global nitrogen cycle [5,6].

Legume–rhizobium symbioses are usually initiated by the exchange of chemical signals between the host and the bacteria. Under low-nitrogen conditions, legumes secrete specific flavonoids into the rhizosphere [3], which stimulate soil-dwelling rhizobia to produce lipid shell oligosaccharide signal compounds called Nod factors (NFs) [7,8]. NFs initiate the root infection process and induce nodule formation in legume roots [9]. Receptor protein kinases in the roots trigger complex signal cascade reactions after sensing and recognizing NFs [10,11,12]. These processes jointly regulate the expression of early nodulation genes in the root epidermis, ultimately coordinating infection by rhizobia and cortical cell division to form root nodule primordia, which develop into mature nodules with a high nitrogen-fixation capacity. 

Phytohormones, especially auxin, play important roles in establishing legume nodule symbiosis signals [13]. The auxin influx transporter (AUX1/LAX) and auxin efflux promoter (PIN) generate a gradient of auxin throughout the root, which peaks at the quiescent center [14]. The uneven distribution of auxin influences cell division, elongation, and differentiation, driving the formation of the lateral roots and nodules [15,16]. The signals of the auxin response reporter gene GH3: GUS are present in the outer cortical cells in young developing nodules and in vascular tissues in mature nodules, providing further evidence that auxin is associated with nodule formation [17]. Flavonoids also play a crucial role inside the plant root during nodulation. Flavonoids are a group of ubiquitous and diverse molecules produced via the phenylpropanoid pathway in higher plants. A major role thought to be played by flavonoids once the bacteria enter the plant is the modulation of auxin transport during the initiation of nodule primordia [18].

Peanut (*Arachis hypogaea*) is a leguminous species yielding one of the most important oil crops in the world. However, its nodulation process is less well studied than those of soybean (*Glycine max*), *Medicago truncatula*, and *Lotus japonicus*. In *Medicago truncatula*, nods potentiate the transcriptional regulation of the auxin signal pathway. Accordingly, Epidermal auxin biosynthesis facilitates rhizobial infection and restricts cytokinin signaling in *Lotus japonicus* [19,20,21]. Unlike the bacterial infection lines observed in soybean and alfalfa, rhizobia enter peanut roots through cracks around the bases of the lateral roots [22]. The molecular mechanisms that affect peanut–rhizobia symbioses may therefore differ from those of other legumes. In the present study, transcriptomic and metabolomic analyses of non-nodulating plants and the nodulating cultivar Huayu22 were carried out to explore the nodulation mechanism of peanut. Improved genomic resources may enhance the current understanding of the mechanisms of rhizobial nitrogen fixation in peanut. 

## 2. Results

### 2.1. Phenotypic Variations between Peanut Plants That Do Not Nodulate and Those That Do

**Figure 1:**
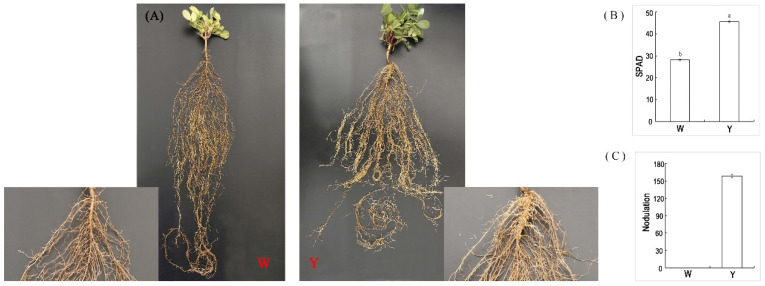
Phenotypes of W and Y plants grown under the same conditions. (**A**) Whole-plant phenotype and root nodule detail. (**B**) SPAD in leaves. (**C**) Number of nodules per plant. Error bars indicate SE of at least three biological repeats. Different letters indicate a significant difference between W and Y plants, as determined using Student’s *t*-test.

Under the same cultivation conditions, non-nodulating (W) and nodulating (Y) peanut plants exhibited significant phenotypic differences (Figure 1). The aerial parts of the Y plants grew vigorously, with dark-green leaves, whereas those of the W plants grew weakly and had yellow-green leaves. The soil and plant analyzer development (SPAD) value represents the relative value of the chlorophyll content of the leaves and how green the plant is (Figure 1B). The W plants did not have nodules and the Y plants had more than 150 nodules per plant (Figure 1C). 

### 2.2. Differentially Expressed Genes (DEGs) in the Roots of W vs. Y Plants

We performed transcriptome sequencing of the roots of the W and Y plants. We performed three independent biological replicates per genotype, and thus six libraries were constructed and sequenced, resulting in 43.69 million to 47.91 million raw reads per sample (Table 1). After removing the adapter sequences and low-quality reads, we obtained 264.5 million (95.83%) clean reads, representing an average of 44.08 million clean reads per sample. For each sample, over 96% of the clean reads were mapped to the reference genome, and over 82% of reads were matched to a unique location in the reference genome. The density distribution of the expected number of fragments per kilobase of transcript sequence per million base pairs sequenced (FPKM) (Figure S1) and the gene expression distribution box diagram (Figure S2) suggested that the detected genes followed a standard normal distribution, indicating that the sequenced data were of high quality and showed reasonable reproducibility.

**Figure 2:**
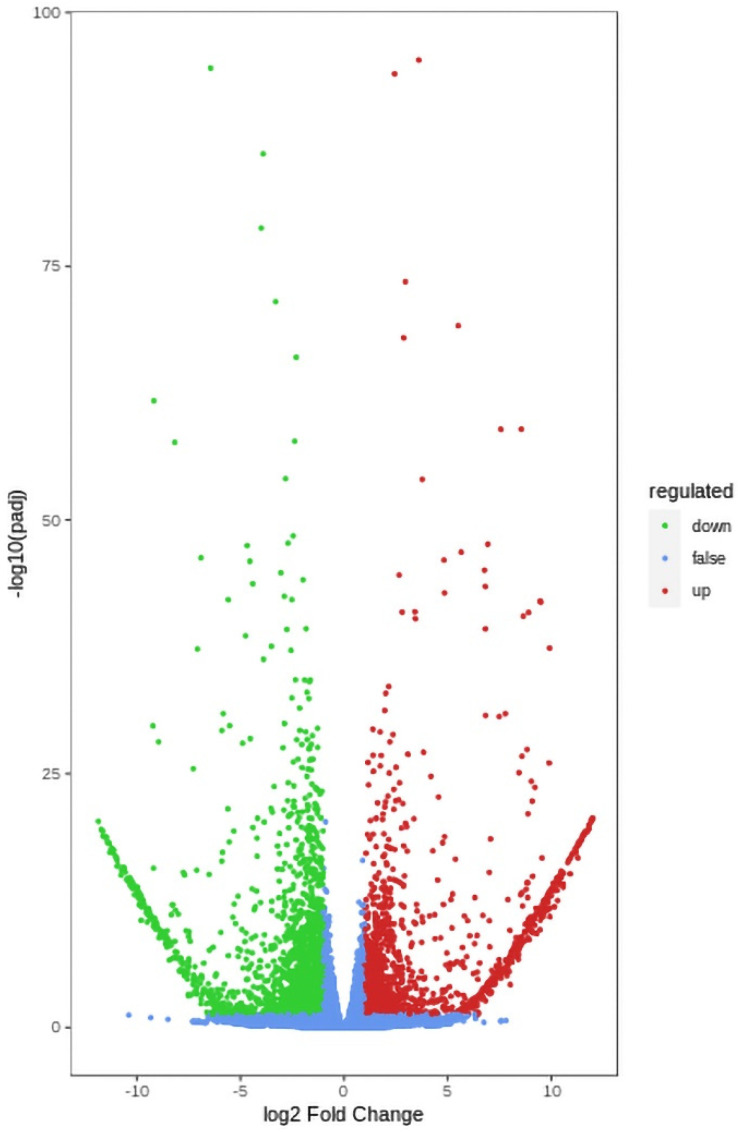
Transcriptome comparison between roots of W and Y plants.

Using the filter criteria |Log_2_ fold change (FC)| > 1 and a false discovery rate (FDR) < 0.05, we identified 3362 DEGs between the W and Y plants roots. A total of 1431 genes were upregulated and 1931 genes were downregulated in the Y plants compared with the W plants (Figure 2 and Table S1).

### 2.3. Gene ontology (GO) and Kyoto Encyclopedia of Genes and Genomes (KEGG) Enrichment of DEGs

**Figure 3:**
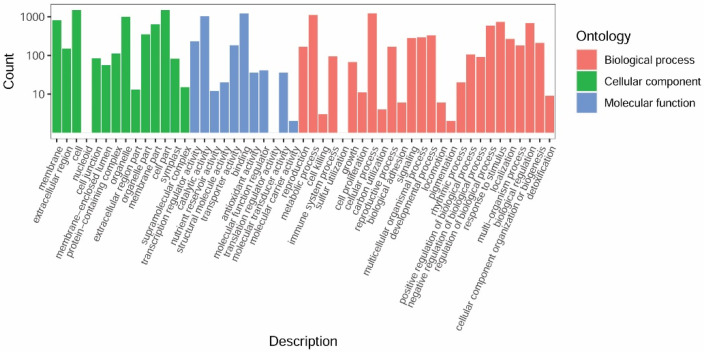
Top 50 gene ontology (GO) terms enriched in the differentially expressed genes (DEGs) between roots of W and Y plants.

Using Blast2GO, 3072 of the 3362 DEGs were assigned to GO terms and were visualized using the WEGO2.0 web-based tool in three main categories: cellular component, molecular function, and biological process (Figure 3). ‘Metabolic process’ and ‘cellular process’ were the most highly represented terms in the biological process category, implying that the metabolic activity and the cellular substances differed between the roots of the non-nodulating and nodulating peanut plants. In the molecular function category, the DEGs were mostly contained within two terms ‘transporter activity’ and ‘catalytic activity’. These results emphasize the potential contribution of substance transport in peanut nodulation.

**Figure 4:**
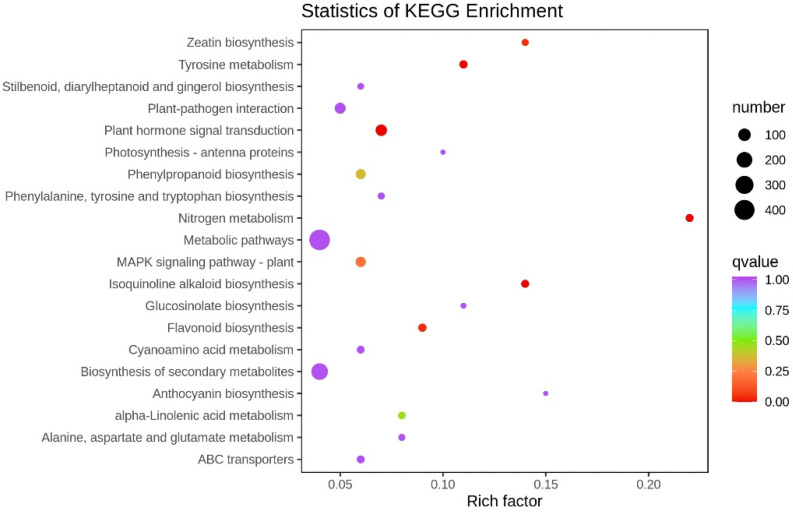
Top 20 Kyoto Encyclopedia of Genes and Genomes (KEGG) pathways enriched in the differentially expressed genes (DEGs).

To explore their biological functions further and to examine their gene interactions, we assigned 906 of the 3362 DEGs to KEGG pathways. Using the criterion *q* < 0.05, 20 KEGG pathways were enriched in the roots of nodulating vs. non-nodulating plants (Figure 4), particularly in activities associated with metabolic pathways, the biosynthesis of secondary metabolites, hormone signal transduction, and ABC transport, as well as zeatin, flavonoid, and phenylalanine biosynthesis. These results suggest that the biosynthesis of various substances influences peanut nodulation.

### 2.4. Widely Targeted Metabolic Profiling Assay in the Roots of W and Y Plants

**Figure 5:**
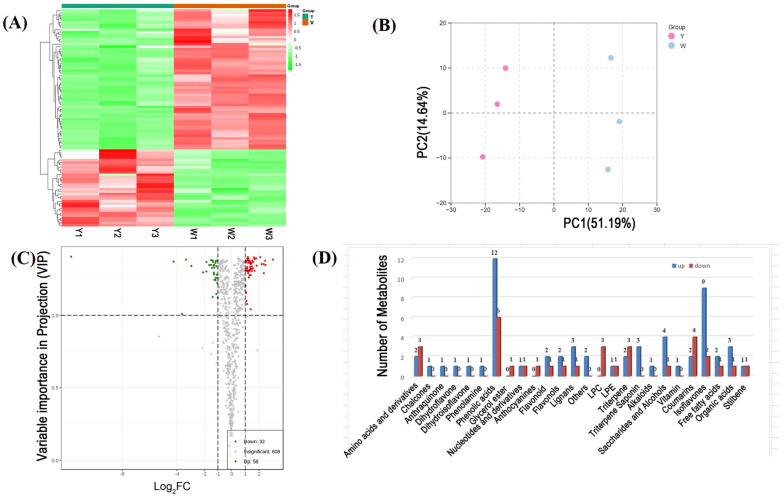
Heat map visualization and principal component analysis (PCA) of the differentially abundant metabolites between the roots of W and Y plants. (**A**) Heat map visualization of the content of each metabolite normalized to the complete linkage hierarchical clustering. Each sample is shown in a single column and each metabolite is represented by a single row. (**B**) Score plots for principal components 1 and 2 between W and Y plants. (**C**) Volcano plot showing the differentially abundant metabolites in W vs. Y plants. (**D**) Number of differentially abundant metabolites between the roots of W and Y plants.

To elucidate the mechanism of nodule formation in peanut, we performed a widely targeted metabolomics assay using the roots of W and Y plants and identified 698 metabolites (Figure 5 and Table S2). A principal component analysis (PCA) showed that these metabolites were divided into two major principal components, indicating that the root metabolites of the two peanut varieties were different. The abundance of 90 metabolites was significantly different in the roots of the W vs. the Y plants, including flavones, isoflavones, organic acids, phenolic compounds, and amino acids. The abundance of 58 metabolites was increased and that of 32 metabolites was decreased in the roots of the Y plants compared with those in the roots of the W plants. Flavonoids were significantly more abundant in the Y plants, including anthocyanins, flavonoids, flavonols, and isoflavones. In particular, the contents of galloylisorhamnetin and formononetin-7-O-(6″-malonyl) glucoside were 1.69- and 2.1-fold greater, respectively, in the Y plants than that in the W plants.

### 2.5. Integrative Analysis of Transcriptomic and Metabolomic Data

**Figure 6:**
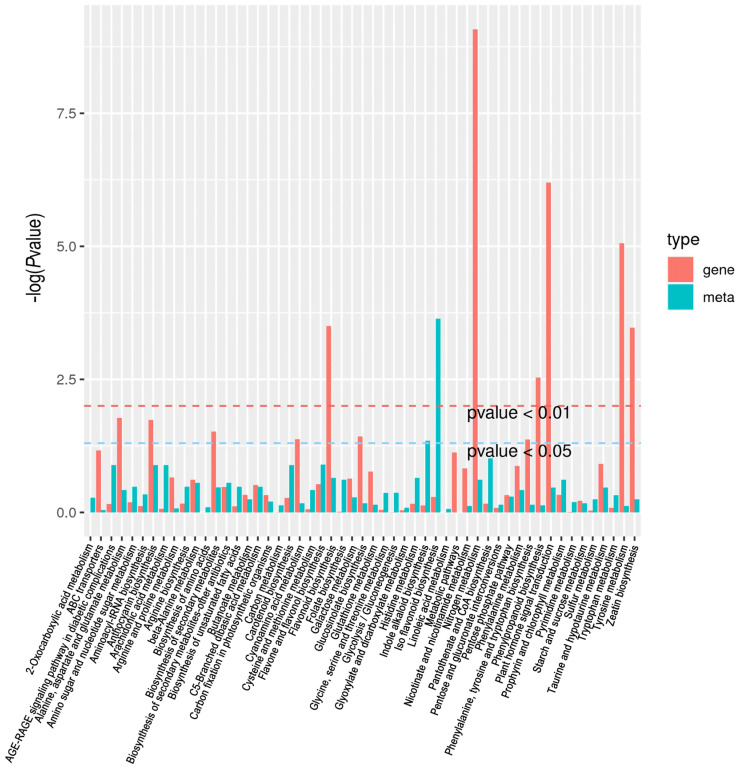
Kyoto Encyclopedia of Genes and Genomes (KEGG) enrichment analysis of the integrated metabolomics and transcriptomics data.

To examine the association between the metabolites and genes involved in a particular biological process, we performed a comprehensive analysis of the metabolomic and transcriptomic data, identifying 53 pathways that were highly differentially expressed pathways in the roots between Y and W plants (Figure 6). These pathways, including flavonoid, isoflavone, phenylpropanoid, zeatin, and secondary metabolite biosynthesis, as well as plant hormone signal transduction, play important roles in legume nodulation [23,24].

In the phenylpropanoid, flavonoid, and isoflavone biosynthetic pathways, we identified 81 DEGs and 12 differentially abundant metabolites. Although the genes encoding isoflavone reductase, flavonoid synthase, chalcone synthase, isoflavone methyltransferase, naringenin oxygenase, and anthocyanin oxygenase were downregulated in the W plants, the total flavonoid content was higher in the W plants than in the Y plants (Table S2).

Genes in plant hormone signal transduction pathways were also differentially expressed between the Y and W plants. Of the 75 representative DEGs in these pathways, 19 were associated with auxin, most of which were downregulated in the W plants. An analysis of the auxin contents in the roots of the W and Y plants showed that the auxin content was significantly reduced in W vs. Y plants (Table 2). This finding suggests that auxin and its signal transduction pathway function in the formation of peanut nodules.

### 2.6. Validation of DEGs Using RT-qPCR

**Figure 7:**
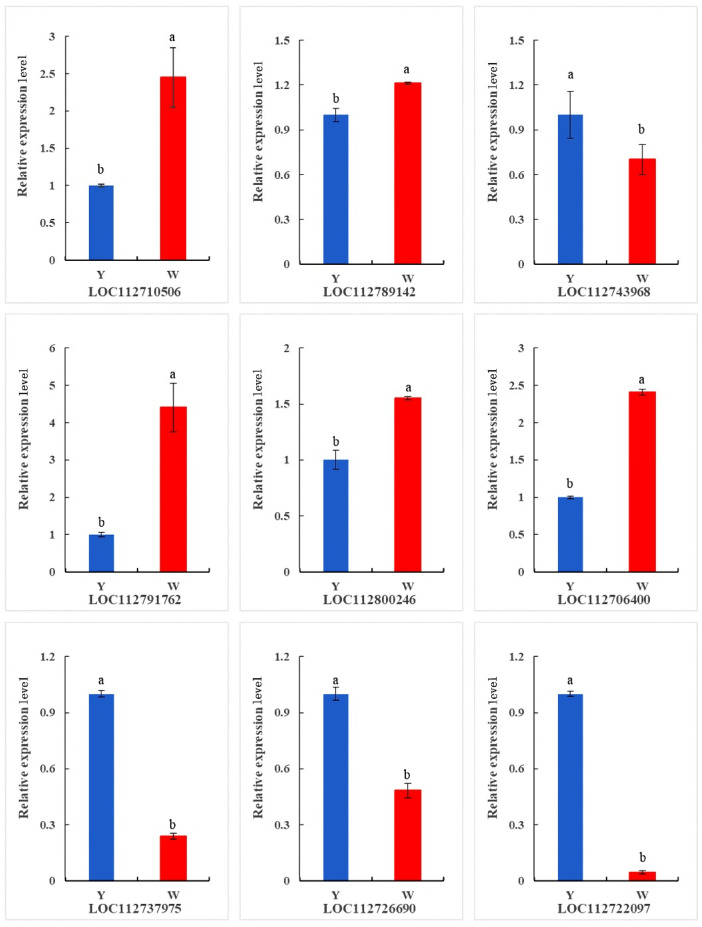
RT-qPCR verification of selected differentially expressed genes (DEGs) of W and Y plants. Three independent repetitions were performed. Different letters indicate a significant difference at *p* ≤ 0.05, as determined using Student’s *t*-test.

We validated the transcriptomic data by analyzing the expression of nine randomly selected DEGs using reverse-transcription quantitative PCR (RT-qPCR). The results showed that five DEGs were more highly expressed in the W than in the Y plants, while four DEGs were upregulated in the Y plants. The consistency between the RNA-seq and RT-qPCR data confirmed the reliability of the transcriptome sequencing results (Figure 7). 

### 2.7. Determination of Auxin Content

Our pathway analysis of the transcriptomic data revealed that many DEGs were involved in hormone signal transduction. Several plant hormones, including auxin, cytokinin, and ethylene, are involved in legume nodulation [25]. To explore the relationship between peanut nodulation and phytohormones, we identified the expression patterns of 75 DEGs from 3362 DEGs involved in hormone signal transduction, including 19 regulators of auxin signaling, 3 regulators of cytokinin, and 8 regulators of ethylene signaling (Table S1).

**Figure 8:**
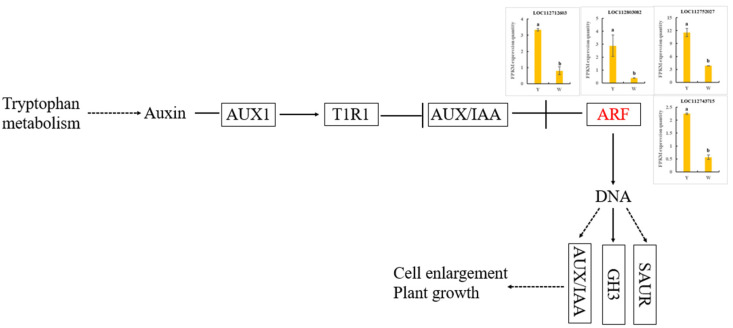
Detection of *ARF* gene expression, a key gene in auxin synthesis pathway.

We further explored the role of auxin in peanut nodulation by measuring the contents of 11 auxin compounds in the roots of the W and Y plants using ultra-performance liquid chromatography (UPLC)–tandem mass spectrometry (MS/MS). Auxin compounds were less abundant in the W than in the Y plants, except for indole-3-acetonitrile (IAN) and indole-3-pyruvic acid (IPA; Table 2). The total auxin content was 5628.77 mg/g in the non-nodulating plants and 6888.59 mg/g in the nodulating plants. We also examined the expression patterns of four auxin response factor (*ARF*) genes. All four genes were expressed with higher levels in the Y plants than in the W plants (Figure 8).

## 3. Discussion

Plant metabolomes are diverse, bridging the gap between genotypes and phenotypes [26,27]. In peanut, phenolic acids, amino acids and their derivatives, and flavonoids have chemotactic effects on rhizobia [28]. In the present study, we identified 46 such compounds among 90 differentially abundant metabolites between non-nodulating (W) and nodulating (Y) peanut varieties (Figure 5D and Table S2), the majority of which were more abundant in W plants. The abundance of these metabolites might attract more rhizobia to the root system of W plants; however, only rhizobia of a specific species can become symbionts in peanut root nodules, limiting the impact of this effect on peanut nodulation.

A subgroup of flavonoids, isoflavones, can induce the expression of nodulation genes by bacteria in the legume rhizosphere. Daidzein and genistein are important isoflavones in legumes [24] and are mainly biosynthesized through the phenylpropionic acid pathway. The cytochrome p450-dependent monooxygenase CYP73 family [29] and chalcone synthase (CHS) [30] are the key enzymes in isoflavone biosynthesis. Through our transcriptomic analysis, we identified two cinnamic acid-4-oxygenase genes (CYP73 family members) and three *CHS* genes (Table 1) that were expressed at low levels in W plants, likely limiting the content of genistein and, thereby, the activation of bacterial nodulation genes.

Although legumes produce a variety of flavonoids, only specific subsets play a role in nodulation [31,32]. In addition to isoflavones, we also identified significant differences in the abundance of flavonols, flavones, and anthocyanins between W and Y plants (Figure 5D and Table S2). In the W plants, quercetin and biotin A were significantly more abundant (Table S2), both of which attract peanut-symbiotic rhizobia [28]. This finding appeared to contradict the observed non-nodulating phenotype of the W plants, leading us to investigate further. 

As a nodule signal, flavonoids must be secreted into the rhizosphere to induce the expression of nodulation genes in bacteria [18]. Thus, the production and release of flavonoids is key to achieving host–symbiont specificity. Through our transcriptomic analysis, we identified four differentially expressed isoflavone-7-O-methyltransferase genes between W and Y plants (Table S2). O-methylation can improve the structural stability, protein affinity, and transport capacity of flavonoids [33,34,35]; thus, the decreased isoflavone-7-O-methyltransferase expression in W plants might reduce the stability and export of isoflavones. We also identified 13 ABC transporter genes (Table S2), which are also involved in flavonoid transport [36]. These transcriptomic differences are expected to affect the signal exchange between peanut and rhizobia such that bacterial nodulation genes are not activated, ultimately preventing peanut nodulation. Flavonoids act as inhibitors during auxin transport in the roots of indeterminate nodule-forming plants, such as white clover and vetch [18]. However, the isoflavone-mediated rhizobia infection site and auxin transport regulation play an important role in peanut nodulation.

The total auxin content was significantly lower in the roots of the W plants than in those of the Y plants (Table 2). Five *AUX/IAA* genes and five auxin response factor (*ARF*) genes, key regulators of the auxin response [37,38], were significantly downregulated in the W plants compared with the Y plants (Table S2). Low auxin concentrations cause the ARF activation factor and AUX/IAA protein to form a heterodimer, which inhibits the activation of auxin-responsive genes. After rhizobial infection, auxin signal transduction in W plants is blocked, preventing the formation of nodules. In addition, ARFs may affect the expression of nodule-associated genes. Silencing *MtARF2*, *MtARF3*, *MtARF4a*, and *MtARF4b* in *Medicago truncatula* alters the mRNA abundance of *early NODULATION SIGNALING PATHWAY 2* (*MtNSP2*) [39]; however, it remains unclear whether peanut ARFs can regulate *NSP* expression to influence nodulation.

## 4. Materials and Methods

### 4.1. Plant Materials

Seeds of Huayu22 (Y) and non-nodulating (W) peanut plants were seeded with rhizobia *Bradyrhizobium yuanmingense* before being planted into 30 cm × 25 cm pots holding a sterilized mixture of 3:1 quartz sand: vermiculite, which was enhanced with a base nutrient solution and fertilizer comprising phosphate (180 kg/ha), potassium (180 kg/ha), and calcium (150 kg/ha). *Bradyrhizobium yuanmingense* belongs to the slow-growing rhizobium CCBAU21353 isolated from peanut root nodule and supplied by Nanjing Normal University. The peanut seeds of Huayu22 (Y) and non-nodulating (W) plants were supplied by Shandong Peanut Research Institute. The rhizobium was cultured in YMA (yeast mannitol agar) medium with OD value of 0.6; meanwhile, the seeds were sterilized with 70% ethanol and washed twice with sterilized water, then immersed in the rhizobia for 4 h and planted in pots. The plants were cultivated under field pot conditions at the Shandong Academy of Agricultural Sciences Station (117°5′ E, 36°43′ N), Jinan, China. Four seeds were planted in each pot and thinned to two plants per pot after emergence. The plants were watered with nutrient solution every 4 days. The roots were taken 45 days after inoculation. The sediment around the sampled roots was washed away with water, and the roots were rinsed with distilled water to prevent sample contamination. After drying, the samples were placed in liquid nitrogen and stored frozen until needed for analysis. Three replicates were used for each treatment. The chlorophyll meter SPAD-502 Plus measures the relative chlorophyll content or “greenness” of plants in real time. The relative content of chlorophyll in the leaves was assessed by measuring the absorption rate of the leaves in the two wavelength bands.

### 4.2. RNA Isolation, cDNA Library Construction, and Sequencing

Total RNA was extracted from frozen roots using the RNAprep Pure Plant Kit (Tiangen Biotech, Beijing, China), and its concentration was measured using a Qubit RNA Assay Kit and Qubit 2.0 Flurometer (Thermo Fisher Scientific, Waltham, MA, USA). The RNA integrity was assessed using an RNA Nano 6000 Assay Kit for the Bioanalyzer 2100 system (Agilent Technologies, Santa Clara, CA, USA). A total of 3 g RNA per sample was prepared for sequencing. Fragmentation was carried out using divalent cations in NEBNext First Strand Synthesis Reaction Buffer (5X; New England Biolabs, Ipswich, MA, USA). First-strand cDNA was synthesized using random hexamer primers and M-MuLV Reverse Transcriptase (RNase H^−^). Second-strand cDNA synthesis was subsequently performed using DNA Polymerase I and RNase H. Any remaining overhangs were converted into blunt ends using exonuclease/polymerase. After the adenylation of the 3′ ends of the cDNA fragments, a NEBNext Adaptor (New England Biolabs) with a hairpin loop structure was ligated to each sample to prepare for hybridization. To select cDNA fragments that were preferentially 150–200 bp in length, the library fragments were purified with the AMPure XP system (Beckman Coulter, Brea, CA, USA). A 3 µL aliquot of SER enzyme (New England Biolabs) was added to each sample and they were incubated at 37 °C for 15 min followed by 5 min at 95 °C. The PCR was performed using Phusion High-Fidelity DNA polymerase, universal PCR primers, and Index (X) Primer. The PCR products were purified (AMPure XP system, Beckman Coulter), and the library quality was assessed on the Bioanalyzer 2100 system (Agilent Technologies). Finally, the cDNA libraries were sequenced using the Illumina HiSeq 4000 platform.

### 4.3. RNA-seq Data Analysis and Annotation

A GO enrichment analysis of the DEGs was implemented in the GO seq R package, correcting for gene length bias. GO terms with a corrected *p* value < 0.05 were considered significantly enriched among the DEGs. The KEGG database is a resource for understanding the high-level functions and utilities of a biological system, such as a cell, organism, or ecosystem, from molecular-level information, especially large-scale molecular datasets generated through genome sequencing and other high-throughput experimental technologies (http://www.genome.jp/kegg/) accessed 5 June 2021. KOBAS software 3.0 was used to test the statistical enrichment of the KEGG pathways among the DEGs.

### 4.4. Extraction of Metabolites

Peanut roots were placed in a freeze-drying machine (Scientz-100F; Ningbo Scientz Biotechnology, Ningbo, China) for freeze-drying and ground to a powder using a grinder (MM 400; Retsch, Haan, Germany) at 30 Hz for 1.5 min. A 100 mg aliquot was dissolved in 1.2 mL 70% methanol and left overnight at 4 °C, vortexed six times throughout the extraction period. After centrifugation at 12,000 rpm for 10 min, the supernatant was extracted and filtered through a 0.22 μm microporous membrane for UPLC–MS/MS analysis. Principal component analysis (PCA) is a technique for simplifying data sets. It is a linear transformation. This transformation transforms the data into a new coordinate system such that the first variance of any data projection is in the first coordinate (called the first principal component), the second variance is in the second coordinate (the second principal component), and so on.

### 4.5. UPLC–MS/MS Analysis

The liquid phase was performed on a Nexera X2 UPLC system (Shimadzu, Kyoto, Japan) using an Agilent SB-C18 chromatographic column (Agilent Technologies; 1.8 μm, 2.1 mm × 100 mm). The mobile phase A was ultra-pure water with 0.1% formic acid, while phase B was acetonitrile with 0.1% formic acid. The elution gradient was as follows: 0 min: 95% phase A, 5% phase B; 0–9 min: phase B ratio increased linearly to 95%; 9–10 min: phase B maintained at 95% for 1 min; 10–11 min: phase B ratio decreased to 5%; and 11–14 min: phase B maintained at 5% for 3 min. The flow rate was 0.35 mL/min, the column temperature was 40 °C, and the injection volume was 4 μL. 

MS was performed using an electrospray ionization temperature of 550 °C, a MS voltage of 5500 V (positive mode)/−4500 V (negative mode), voltage gas at 25 psi, and a high collision-activated dissociation parameter. In the triple quadrupole (QQQ), each ion pair was scanned and detected according to the optimized declustering potential and collision energy [40].

### 4.6. RT-qPCR

Total RNA was reverse-transcribed using a Quantscript reverse transcriptase kit (Tiangen Biotech), and the resulting cDNA was subjected to qPCR using the gene-specific primers shown in Table S3. The RT-qPCR was conducted using an ABI Prism 7500 Real-Time PCR system (Thermo Fisher Scientific) using SYBR Premix Ex Taq II (Takara Bio, Kusatsu, Japan). The comparative 2^–ΔΔCT^ method [41] was used to quantify gene expression relative to peanut reference gene Actin 11.

### 4.7. Auxin Measurement

A 50 mg aliquot of each ground root sample was combined with 10 μL mountain concentration of 100 ng/mL mixed internal standard test solution and 1 mL methanol/water/formic acid (15:4:1, *v*/*v*/*v*) extraction agent. The samples were mixed by vortexing for 10 min and subjected to centrifugation at 12,000 rpm and 4 °C for 5 min. The supernatant was transferred into a new centrifuge tube. After concentration, the sample was mixed with 100 μL 80% methanol/water solution, filtered through a 0.22 μm membrane, and placed in an injection bottle for UPLC–MS/MS analysis.

### 4.8. Statistical Analysis

Statistical analyses were performed via analysis of variance (ANOVA) using SPSS version 13.0 (SPSS, Chicago, IL, USA) and the differences were considered statistically significant at *p*-value less than 0.05. The values of SPAD, nodulation, and the relative expression level were used for the statistical analyses.

## 5. Conclusions

The important roles of flavonoids and auxin in peanut nodulation were elucidated by our combined transcriptomic and metabolomic analyses. Twenty genes involved in flavonoid biosynthesis and transport were identified; the low expression of these genes could limit flavonoid contents and likely inhibit the transfer of flavonoids from the plant root to the rhizosphere (soil). In addition, 10 genes encoding the ARF and AUX/IAA proteins were significantly downregulated in the non-nodulating W plants compared with the nodulating Y plants. Our comprehensive analysis of the transcriptomic and metabolomic data indicated that non-nodulating plants do not transfer rhizobia-attracting flavonoids into the rhizosphere, preventing the bacteria from recognizing the peanut roots and expressing their nodulation-promoting genes. Furthermore, the low expression of ARF-encoding genes in W plants affects their auxin signal transduction and the resulting formation of peanut nodules. Our research results provide a basis for improving peanut nodulation efficiency.

## Figures and Tables

**Table 1:** Quality statistics of the sequencing output.

Sample	Raw Reads	Clean Reads	Clean Bases (Gb)	Error Rate (%)	Q20 (%)	Q30 (%)	GC Content (%)	Reads Mapped	Unique Mapped	Multi Mapped
W1	43,695,792	42,125,346	6.32	0.03	97.86	93.83	43.66	40,827,293 (96.92%)	36,659,116 (84.65%)	6,532,025 (12.52%)
W2	47,912,986	46,155,764	6.92	0.03	97.86	93.84	43.94	44,736,326 (96.92%)	38,879,339 (84.24%)	7,145,385 (12.69%)
W3	44,948,048	43,304,136	6.5	0.02	98.06	94.32	43.96	42,182,757 (97.41%)	36,659,116 (84.65%)	6,801,200 (12.76%)
Y1	47,519,936	45,683,364	6.85	0.02	98.04	94.27	43.99	44,524,465 (97.46%)	3,8478,645 (84.23%)	
Y2	47,099,980	45,145,906	6.77	0.02	98.05	94.30	43.77	43,799,436 (97.02%)	37,955,896 (84.07%)	
Y3	44,867,488	42,092,504	6.31	0.03	97.95	94.06	43.79	40,907,322 (97.18%)	34,907,249 (82.93%)	

Raw reads: Total number of reads obtained before filtering; Clean reads: Number of reads remaining after filtering; Clean bases (Gb): Total number of bases after filtration; Q20 (%): Proportion of nucleotides with a quality value larger than 20 in the filtered reads; Q30 (%): Proportion of nucleotides with a quality value larger than 30 in the filtered reads.

**Table 2:** The contents of endogenous auxin compound in the roots of W and Y plants. Three independent repetitions were performed. Different letters indicate a significant difference between W and Y at *p* ≤ 0.05.

Auxin (mg/g)	W	Y
ICA	5.74 a	6.98 b
ICAld	11.05 a	10.92 a
IAA	11.03 a	34.92 b
IAN	0.3 a	0.26 a
IAA-Gly	3.01 a	2.82 a
IAA-Phe-Me	0.34 a	0.5 a
TRA	0.63 a	0.87 a
IAA-Glu	24.51 a	208.4 b
MEIAA	1.39 a	4.88 b
IPA	8.97 b	4.94 a
TRP	5561.8 a	6613.1 b
Total	5628.77 a	6888.59 b

## Data Availability

The data and materials that were analyzed in the current study are available from the corresponding author upon reasonable request.

## Supplementary Materials

The supporting information can be downloaded at: https://www.mdpi.com/article/10.3390/ijms241210152/s1.
