# Supplementary material for: Transcriptomic and Metabolomic Analyses Reveal the Roles of Flavonoids and Auxin on Peanut Nodulation
Source: Int J Mol Sci. 2023 Jun 15;24(12):10152. doi: 10.3390/ijms241210152 (PMC10299696; doi:10.3390/ijms241210152)
Supplement: Supplementary file 1 [file ijms-24-10152-s001.zip › Figures.pdf]

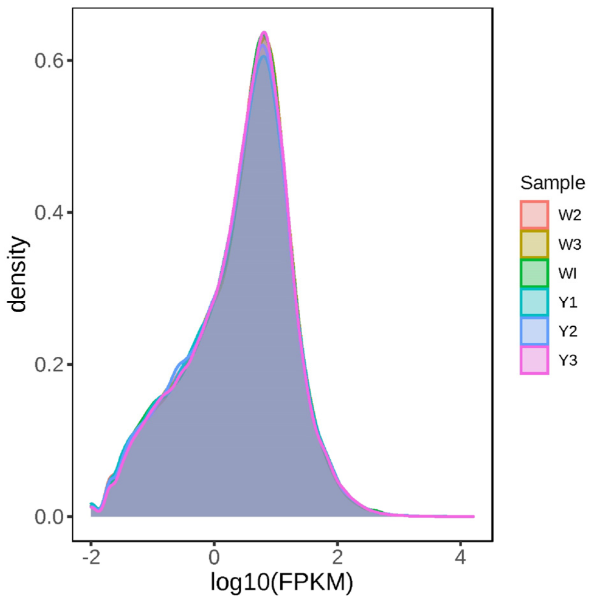

Figure S1 Density distribution of the expected number of fragments per kilobase of transcript sequence per million base pairs sequenced (FPKM) values.

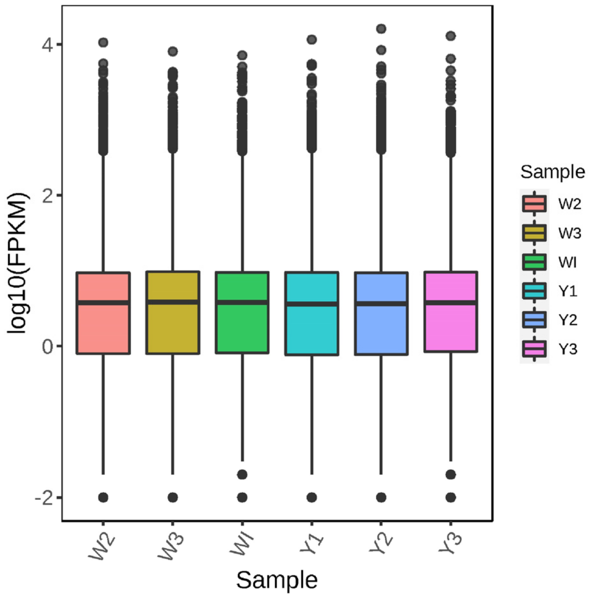

Figure S2 Expected number of fragments per kilobase of transcript sequence per million base pairs sequenced (FPKM) values for each sample. The expression level of each sample was measured from the overall discrete point of view.
